# Omega-3 Polyunsaturated Fatty Acid Attenuates Uremia-Induced Brain Damage in Mice

**DOI:** 10.3390/ijms222111802

**Published:** 2021-10-30

**Authors:** Eun-Ji Kim, Young Rok Ham, Jin Ah Shin, Jin Young Jeong, Ki Ryang Na, Kang Wook Lee, Jwa-Jin Kim, Dae Eun Choi

**Affiliations:** 1 Department of Medical Science, Medical School, Chungnam National University, Daejeon 35015, Korea; kime78715@gmail.com (E.-J.K.); wlsdkahh@gmail.com (J.A.S.); 2 Department of Nephrology, Medical School, Chungnam National University, Daejeon 35015, Korea; youngrok01@cnuh.co.kr (Y.R.H.); spwlsdud@naver.com (J.Y.J.); drngr@cnu.ac.kr (K.R.N.); kwlee@cnu.ac.kr (K.W.L.)

**Keywords:** ω3-PUFA, ischemia-reperfusion, uremic toxin, indoxyl sulfate, brain injury, apoptosis, PI(3)K-Akt signaling

## Abstract

Although the cause of neurological disease in patients with chronic kidney disease (CKD) has not been completely identified yet, recent papers have identified accumulated uremic toxin as its main cause. Additionally, omega-3 polyunsaturated fatty acid (ω-3 PUFA) plays an important role in maintaining normal nerve function, but its protective effects against uremic toxin is unclear. The objective of this study was to identify brain damage caused by uremic toxicity and determine the protective effects of ω-3 PUFA against uremic toxin. We divided mice into the following groups: wild-type (wt) sham (*n* = 8), ω-3 PUFA sham (*n* = 8), Fat-1 sham (*n* = 8), ischemia-reperfusion (IR) (*n* = 20), and ω-3 PUFA+IR (*n* = 20) Fat-1+IR (*n* = 20). Brain tissue, kidney tissue, and blood were collected three days after the operation of mice (sham and IR operation). This study showed that Ki67 and neuronal nuclei (NeuN) decreased in the brain of uremic mice as compared to wt mice brain, but increased in the ω-3 PUFA-treated uremic mice and the brain of uremic Fat-1 mice as compared to the brain of uremic mice. The pro-apoptotic protein expressions were increased, whereas anti-apoptotic protein expression decreased in the brain of uremic mice as compared to wt mice brain. However, apoptotic protein expression decreased in the ω-3 PUFA-treated uremic mice and the brain of uremic Fat-1 mice as compared to the brain of uremic mice. Furthermore, the brain of ω-3 PUFA-treated uremic mice and uremic Fat-1 mice showed increased expression of p-PI3K, p-PDK1, and p-Akt as compared to the brain of uremic mice. We confirm that uremic toxin damages the brain and causes cell death. In these injuries, ω-3 PUFA plays an important role in neuroprotection through PI(3)K-Akt signaling.

## 1. Introduction

It is well known that multiple organs, including the brain, heart, lungs, and liver, are injured/affected progressively in patients with chronic kidney disease (CKD) [1,2]. Although various factors such as acid–base imbalance, volume overload, electrolyte derangement, and accumulation of uremic products cause these damages, uremic toxins are believed to play a major role in the progression of multiple organ damage [3,4]. When kidney clearance decreases, waste nitrogen products and various uremic toxins, including indoxyl sulfate, p-cresol, and hippuric acid, continue to accumulate in blood, thereby causing abnormally high levels. Most of these toxins damage organs and decrease their functions. In particular, they cause neurotoxicity, endothelial damage, and cognitive impairment in the nervous system [5]. Lee et al. reported that uremia increases endoplasmic reticulum (ER) stress and cell death in hippocampus. In addition, uremic toxin increases the reactive oxygen species (ROS) level and transitions to apoptosis in brain cells (neuroblastoma cells) [6]. However, neuronal damage by uremic toxin has not been fully explained, and the underlying explanations for this observation are not clear.

One of the most important uremic toxins is indoxyl sulfate (IS). The serum levels of IS increase when there is a deterioration in kidney function [7]. IS is a protein-bound uremic toxin that is not effectively removed by dialysis. IS is mainly accumulated in the brain stem in a concentration-dependent manner and also is accumulated in the hippocampus [8].

Omega-3 polyunsaturated unsaturated fatty acids (ω3-PUFA) are a group of essential polyunsaturated fatty acids (PUFAs) present in the diet and highly related to the health of the organism [9]. In particular, docosahexaenoic acid (DHA, 22:6, n-3) and eicosapentaenoic acid (EPA), the two main biologically active components of n-3 PUFA, are fats found in many fish, but are not synthesized in the human diet and must be obtained directly [10].

ω3-PUFA is known to play an important role against central nervous system injury via anti-inflammatory effects [11]. Additionally, DHA enrichment protects neurons from apoptotic cell death induced by staurosporine [12]. EPA, a precursor to DHA, has also been shown to exhibit neuroprotective effects from neuronal cell death and apoptosis [13]. Phosphatidylinositol 3-kinase -Akt signals act as networks that regulate cellular responses to external stimuli and metabolic processes, and are important mechanisms for neuronal survival [13,14,15]. DHA, the main component of ω3-PUFA, increases phosphatidylserine in nerve cells, resulting in Akt phosphorylation and activation [16,17]. Similarly, EPA, a precursor to DHA, has also been shown to exhibit neuroprotective effects by increasing the phosphorylation of Akt and inhibiting the activity of caspase-3 [13]. These findings suggested the neuroprotection of ω3-PUFA through PI(3)K-Akt signaling in brain injury.

Although little is known about ω3-PUFA directly reducing brain cell damage caused by uremic toxicity, it has been reported that uremia-induced brain damage is mainly due to an increase in ROS and apoptosis [6]. In addition, omega 3 effectively reduces ROS and apoptosis in various brain injury models [12]. It can be speculated that omega 3 may play a role in reducing brain damage caused by uremia.

Fat-1 transgenic mice express an increase in ω3-PUFAs and reduction in ω6-PUFAs in various tissues, including the muscle, red blood cells, kidney, lung, spleen, heart, brain, and liver [18]. Despite marked change in the ratio of n-6 to n-3, Fat-1 mice are apparently normal and healthy. Thus, these mice can help to clearly determine the effect of omega 3 in brain.

The objective of this study was to examine new molecular factors of uremic toxin that contribute to damage to the brain tissue, identify how ω3-PUFA alleviates brain damage, and evaluate the associated molecular mechanisms.

## 2. Results

### 2.1. Generation of Uremic Status after Ischemia Reperfusion (IR) Renal Injury

**Figure 1:**
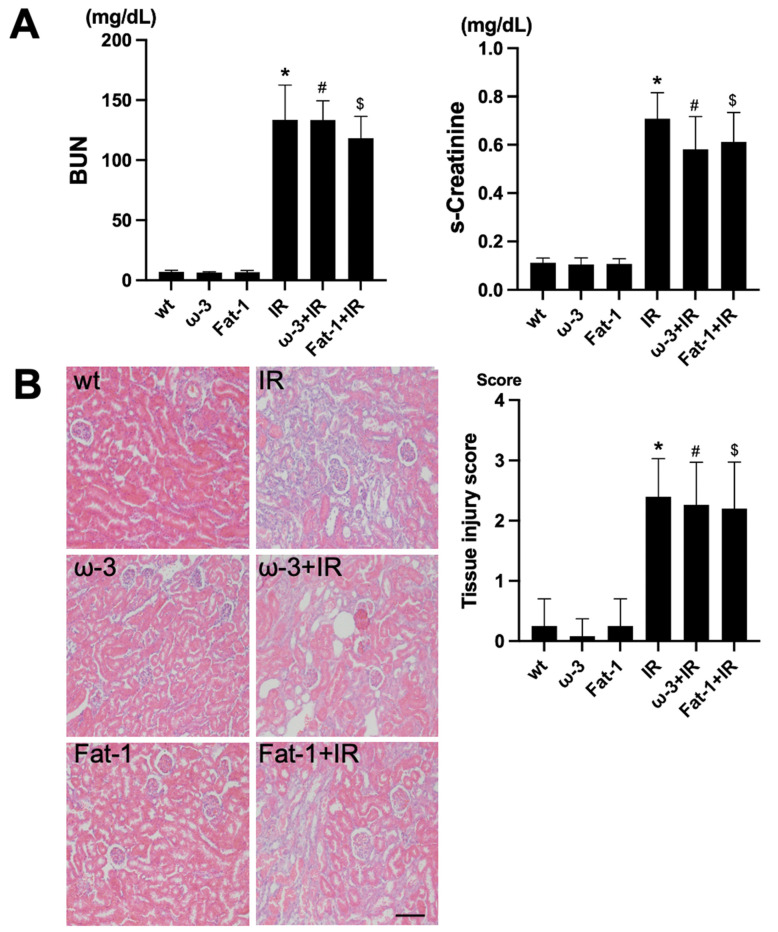
Renal function and histology in ischemia-reperfusion (IR) kidney. (**A**) The levels of blood urea nitrogen (BUN) and serum creatinine (s-Cr) were significantly increased in IR mice compared to wt mice. ω-3+IR mice also had higher BUN and s-Cr levels as compared to ω-3 mice, and Fat-1+IR mice also had higher BUN and s-Cr levels as compared to Fat-1 mice. (**B**) Representative kidney section with H&E stain; renal injury tissue represents cell debris, tubular necrosis, and inflammatory cells. Original magnification, 200×. Scale bar = 50 μm. * *p* < 0.001 vs. wt, # *p* < 0.001 vs. ω-3, $ *p* < 0.001 vs. Fat-1. The bar represents mean ± S.D. (wt, wild-type sham; ω-3, ω-3 PUFA oral administration sham; Fat-1, Fat-1 induction sham; IR, IR renal injury in wild-type mice; ω-3+IR, IR renal injury in ω-3 PUFA oral administration mice; Fat-1+IR, IR renal injury in Fat-1 induction mice).

For the evaluation of renal function, assessment of blood urea nitrogen (BUN) and serum creatinine (s-Cr) was conducted. Comparing blood tests after IR among wt, ω-3, and Fat-1, the levels of BUN and s-Cr were significantly increased after IR injury compared to each control (Figure 1A). H&E staining for histological evaluation revealed renal tubular interstitial damage, including tubular epithelial cell damage and tubular interstitial inflammation in the IR injury kidneys. Similar to the blood test, the renal tubule-interstitial injury was significantly increased in the IR injury kidneys compared to each control (Figure 1B).

### 2.2. Brain Damage in Uremia

**Figure 2:**
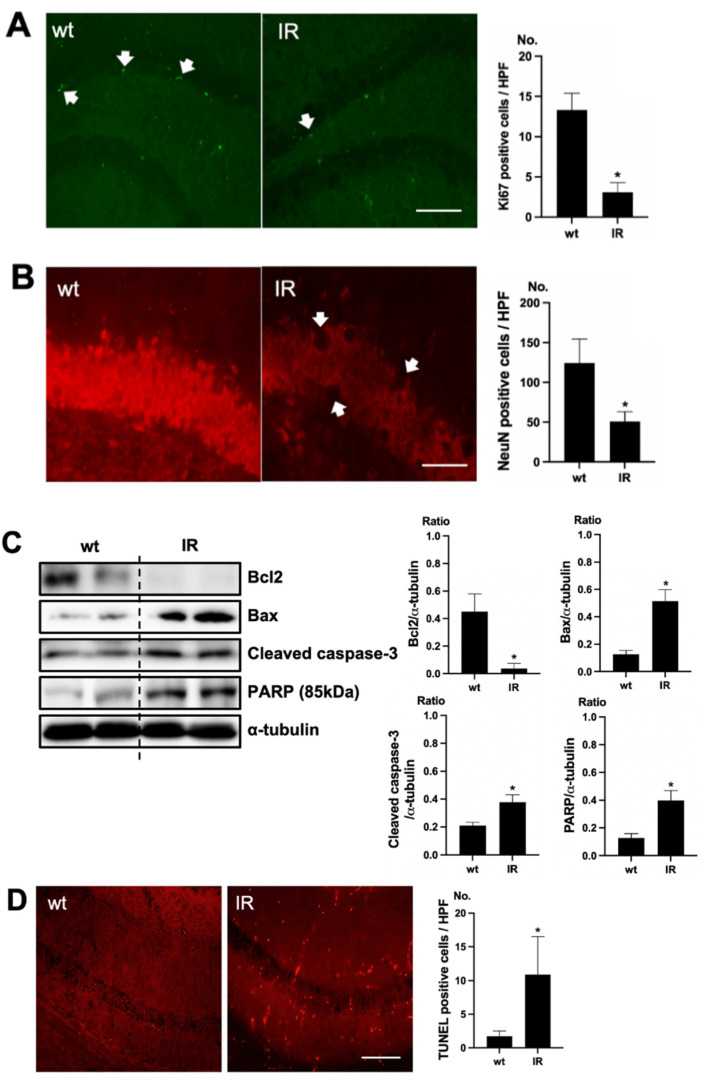
Brain damage in ischemia-reperfusion (IR) kidney. (**A**) Representative immunofluorescence stain of subgranular zone of the hippocampal DG of brain section: Immunofluorescent staining was performed using the markers of proliferation (Ki67) in brain. High power field (HPF), original magnification, 200×. Scale bar = 50 μm. (**B**) Representative immunofluorescence stain of subgranular zone of the hippocampal DG of brain section: Immunofluorescent staining was performed using the neuronal nuclei marker (NeuN) in the brain. Original magnification, 400×. Scale bar = 200 μm. (**C**) Representative Western blot of brain lysis: Western blot analysis shows renal injury—increased Bax, cleaved caspase3 expression, and decreased Bcl2 expression in the brain of uremic mice. Renal injury increased PARP (85 kDa) expression in the brain of uremic mice. (**D**) Representative TUNEL stain of subgranular zone of the hippocampal DG of brain section. Original magnification, 200×. Scale bar = 50 μm. * *p* < 0.001 vs. wt. Bar represents mean ± S.D. (wt, wild-type sham; IR, IR renal injury in wild-type mice).

To verify cell death in the brain of uremic mice, immunofluorescence staining was examined using Ki67 as a proliferating cell marker and NeuN as a neuronal nuclei marker. Positive cells of Ki67 were decreased in the subgranular zone of the hippocampal dentate gyrus (DG) of the brain of uremic mice, compared to the wt mice brain (Figure 2A). Positive cells of NeuN were also decreased in the subgranular zone of the hippocampal DG of the brain of uremic mice, compared to that of wt mice (Figure 2B). For estimating the cell injury in the brain of uremic mice, the expressions of Bcl2, Bax, cleaved caspase-3, and PARP (85kDa) were evaluated. The protein levels of Bax, cleaved caspase-3, and PARP were significantly increased in the brain of uremic mice as compared to wt mice brain, but Bcl2 was significantly decreased in the brain of uremic mice compared to the wt mice brain (Figure 2C). For the evaluation of apoptosis in the brain of uremic mice, neuronal TUNEL-positive cells were evaluated. The numbers of TUNEL-positive cells were higher in the subgranular zone of the hippocampal DG of the brain of uremic mice as compared to that of the wt mice (Figure 2D).

### 2.3. ω-3 PUFA Attenuates Brain Injury in Uremic Mice

**Figure 3:**
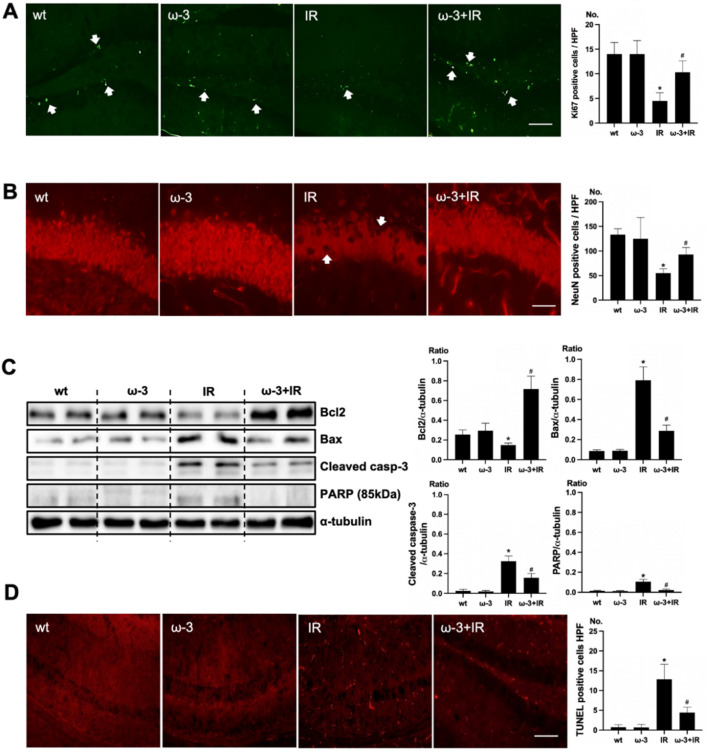
Effects of oral administration ω-3 PUFA on brain damage in ischemia-reperfusion (IR) kidney. (**A**) Representative immunofluorescence stain of subgranular zone of the hippocampal DG of the brain section: Immunofluorescent staining was performed using Ki67 in the brain. High power field (HPF), original magnification, 200×. Scale bar = 50 μm. (**B**) Representative immunofluorescence stain of subgranular zone of the hippocampal DG of brain section: Immunofluorescent staining was performed by using NeuN in the brain. Original magnification, 400×. Scale bar = 200 μm. (**C**) Representative Western blot of brain lysis: The brain of ω-3 PUFA-treated uremic mice showed decreased Bax, cleaved caspase3 expression, and increased Bcl2 expression compared to the brain of uremic mice. Additionally, PARP (85 kDa) expression was also reduced in the brain of ω-3 PUFA-treated uremic mice compared to the brain of uremic mice. (**D**) Representative TUNEL stain of the subgranular zone of the hippocampal DG of the brain section. Original magnification, 200×. Scale bar = 50 μm. * *p* < 0.001 vs. wt, # *p* < 0.001 vs. IR. Bar represents mean ± S.D. (wt, wild-type sham; ω-3, ω-3 PUFA oral administration sham; IR, IR renal injury in wild-type mice; ω-3+IR, IR renal injury in ω-3 PUFA oral administration mice).

To verify the effect of oral administration of ω-3 PUFA on uremic toxin, Ki67 and NeuN was examined. Positive cells of Ki67 were increased in the subgranular zone of the hippocampal DG of the brain of ω-3 PUFA–treated uremic mice compared to that of uremic mice (Figure 3A). Positive cells of NeuN were decreased in the subgranular zone of the hippocampal DG of brain of uremic mice as compared to ω-3 PUFA–treated uremic mice (Figure 3B). Additionally, the protein expressions of Bax, cleaved caspase-3, and PARP were significantly decreased in the brain of ω-3 PUFA–treated uremic mice as compared to the brain of uremic mice. However, Bcl2 was significantly increased in the brain of ω-3 PUFA–treated uremic mice as compared to the brain of uremic mice (Figure 3C). Furthermore, the numbers of TUNEL-positive cells were lower in the subgranular zone of the hippocampal DG of the brain of ω-3 PUFA–treated uremic mice as compared to that of uremic mice (Figure 3D).

### 2.4. Brain Damage Is Attenuated in Fat-1 Mice

**Figure 4:**
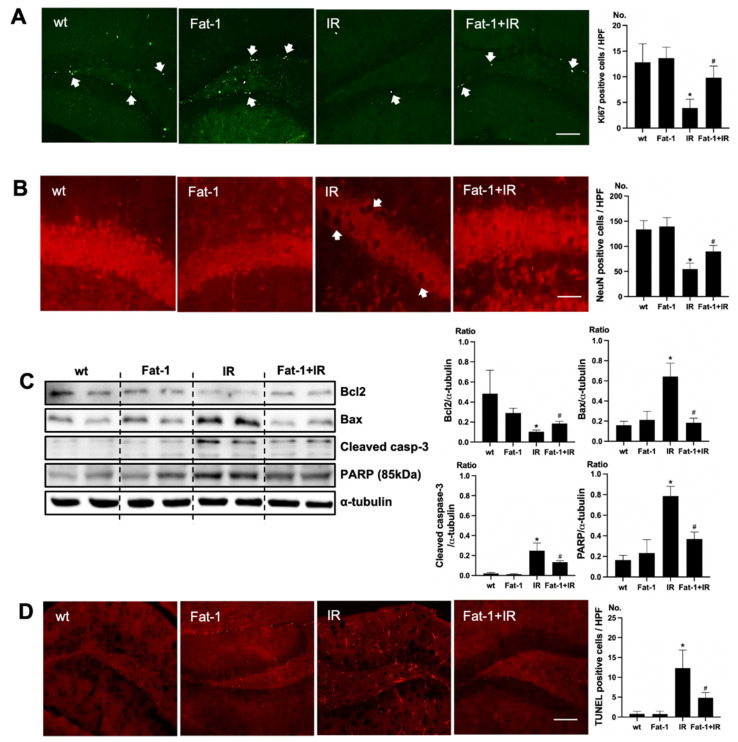
Effects of ω-3 PUFA on brain damage in ischemia-reperfusion (IR) injured Fat-1 mice. (**A**) Representative immunofluorescence stain of the subgranular zone of the hippocampal DG of the brain section: Immunofluorescent staining was performed using Ki67 in the brain. High power field (HPF), original magnification, 200×. Scale bar = 50 μm. (**B**) Representative immunofluorescence stain of the subgranular zone of the hippocampal DG of the brain section: Immunofluorescent staining was performed using NeuN in the brain. Original magnification, 400×. Scale bar = 200 μm. (**C**) Representative Western blot of brain lysis: The brain of uremic Fat-1 mice shows decreased Bax, cleaved caspase3 expression, and increased Bcl2 expression compared to the brain of uremic mice. Additionally, PARP (85 kDa) expression was also reduced in the brain of uremic Fat-1 mice compared to the brain of uremic mice. (**D**) Representative TUNEL stain of the subgranular zone of the hippocampal DG of the brain section. Original magnification, 200×. Scale bar = 50 μm. * *p* < 0.001 vs. wt, # *p* < 0.001 vs. IR. Bar represents mean ± S.D. (wt, wild-type sham; Fat-1, Fat-1 induction sham; IR, IR renal injury in wild-type mice; Fat-1+IR, IR renal injury in Fat-1 induction mice).

In Fat-1 mice, positive cells of immunofluorescence staining of Ki67 were increased in the subgranular zone of the hippocampal DG of brain of uremic Fat-1 mice as compared to that of uremic mice (Figure 4A). Positive cells of immunofluorescence staining of NeuN were increased in the subgranular zone of the hippocampal DG of the brain of uremic Fat-1 mice as compared to that of uremic mice (Figure 4B). Additionally, the protein expressions of Bax, cleaved caspase-3, and PARP were significantly decreased in the brain of uremic Fat-1 mice as compared to the brain of uremic mice. However, Bcl2 was significantly increased in the brain of uremic Fat-1 mice as compared to the brain of uremic mice (Figure 4C). Furthermore, the numbers of TUNEL-positive cells were lower in the subgranular zone of the hippocampal DG of brain of uremic Fat-1 mice as compared to that of uremic mice (Figure 4D).

### 2.5. ω-3 PUFA Activates PI(3)K-Akt Signaling

**Figure 5:**
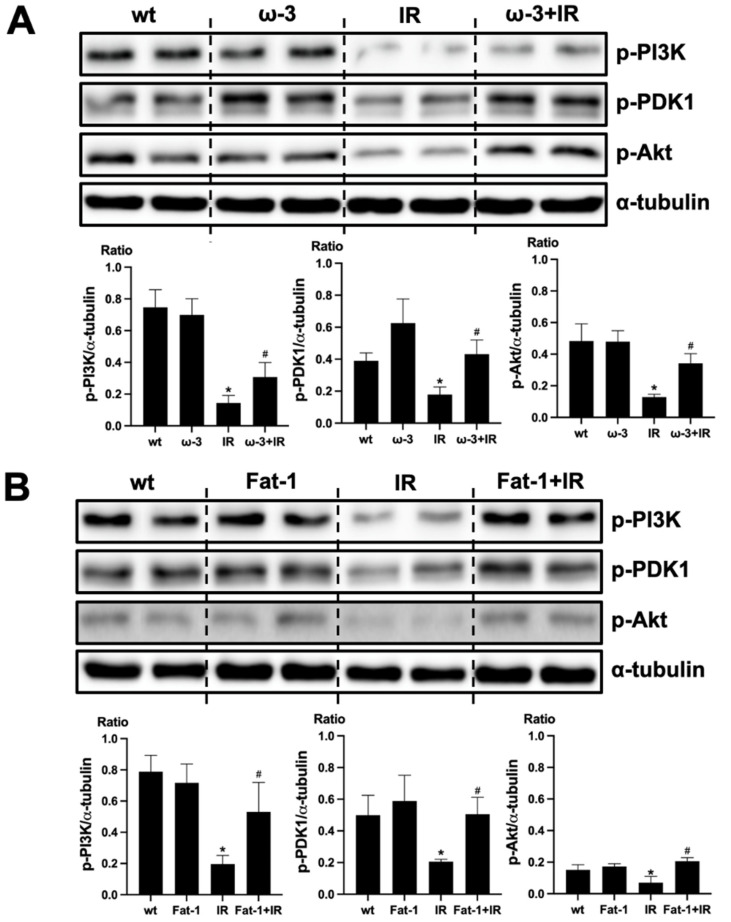
Effects of ω-3 PUFA on PI(3)K/Akt signaling. (**A**) Representative Western blot of brain lysis: The brain of ω-3 PUFA orally administered IR-injured mice shows increased p-PI3K, p-PDK1, and p-Akt expression compared to IR-injured mice. (**B**) Representative Western blot of brain lysis: The brain of IR-injured Fat-1 mice showed increased p-PI3K, p-PDK1, and p-Akt expression, compared to that of IR-injured mice. * *p* < 0.001 vs. wt, # *p* < 0.001 vs. IR. Bar represents mean ± S.D. (wt, wild-type sham; ω-3 PUFA, ω-3 PUFA oral administration sham; Fat-1, Fat-1 induction sham; IR, IR renal injury in wild-type mice; ω-3+IR, IR renal injury in ω-3 PUFA oral administration mice; Fat-1+IR, IR renal injury in Fat-1 induction mice).

We evaluated that PI(3)K-Akt signaling is associated with the protective effect of ω-3 PUFA in the brain injury by uremic toxin. The protein expression of p-PI(3)K, p-PDK1, and p-Akt decreased in the brain of uremic mice compared to that of wt mice brain. ω-3 PUFA treatment significantly increased the protein expression of p-PI(3)K, p-PDK1, and p-Akt in the brain of uremic mice (Figure 5A). Similar to the brain of ω-3 PUFA-treated uremic mice, p-PI(3)K, p-PDK1, and p-Akt protein expressions were significantly increased in the brain of uremic Fat-1 mice as compared to the brain of uremic mice (Figure 5B).

### 2.6. Anti-Apoptotic Effect of ω-3 PUFA in HT22 Cells

**Figure 6:**
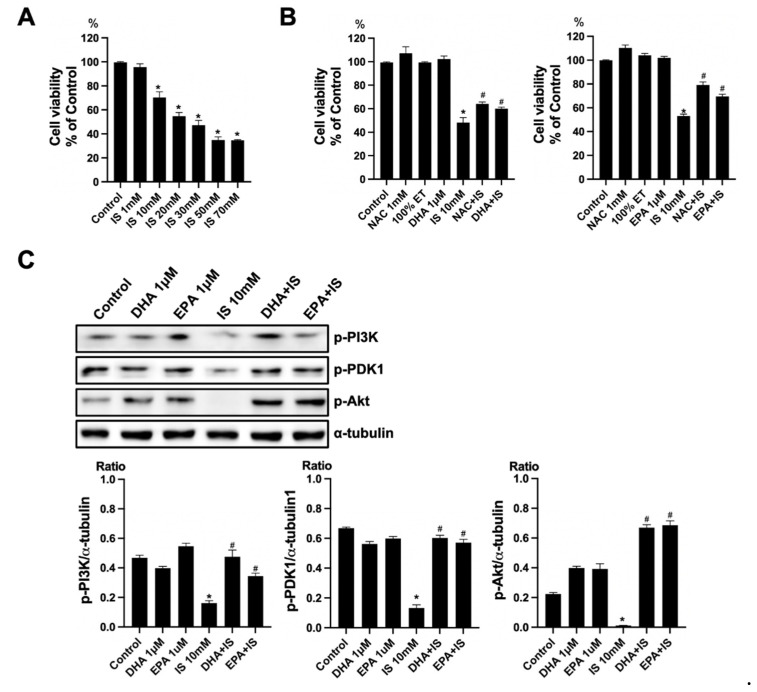
Effects of DHA and EPA on IS toxicity in HT22. (**A**) IS significantly decreased the cell survival in HT22 cells in a dose-dependent manner. (**B**) NAC increased the cell viability in IS-treated HT22 cells. DHA and EPA also increased the cell survival in IS-treated HT22 cells. (**C**) Representative Western blot: IS-treated HT22 cells decreased p-PI3K, p-PDK1, and p-Akt expression. DHA and EPA post-treatment increased p-PI3K, p-PDK1, and p-Akt expression in IS-treated HT22 cells. * *p* < 0.001 vs. control, # *p* < 0.001 vs. IS. Bar represents mean ± S.D. (IS, indoxyl sulfate; NAC, *N*-acetylcysteine; ET, ethanol; MT, methanol; DHA, docosahexaenoic acid; EPA, eicosapentaenoic acid).

To verify the toxicity of IS, the HT22 cells were exposed to various concentrations of IS (1, 10, 20, 30, 50, and 70 mM) for 24 h. The cell viability was determined using a CCK-8 assay. The treatment with IS for 24 h showed a gradual decrease in cell viability at higher concentrations as compared to the control (Figure 6A). To verify the effect of ω-3 PUFA on the toxicity of IS, the HT22 cells were seeded on the plate for 16–18 h. Thereafter, HT22 cells were treated with IS+DHA or IS+EPA for 24 h. As compared with the control, the IS-treated group showed a significant decrease in cell viability. When DHA and EPA were added to the IS-treated group, the cell viability increased significantly compared with the IS-treated group (Figure 6B). Additionally, the protein expression of p-PI(3)K, p-PDK1, and p-Akt decreased in the IS-treated group compared to the control, but when DHA and EPA were added to the IS-treated group, these expressions significantly increased as compared to the IS-treated group (Figure 6C).

## 3. Discussion

Here, we report that ω-3 PUFA protects brain injury by uremic toxin through PI(3)K-Akt signaling. The data showed that uremia decreased neuronal cell proliferation and increased apoptotic protein expression. However, this injury disappeared following treatment of ω-3 PUFA, thereby indicating that ω-3 PUFA was involved in responding to the brain injury. In the future, these results may be used to protect against uremic toxin in brain.

We confirmed the neuronal cell death of the hippocampus region in uremia. In research papers, necrosis and apoptosis increased in the hippocampus CA1 region of the AKI model [19], and in the CKD model, it was confirmed that the ROS level increased in the neuronal cell, thereby leading to cell death [6]. By confirming cleaved caspase-3 and PARP (85 kDa) protein expression, we identified that neuronal cell death in uremia occurs through apoptosis, confirming the results of previous research.

When measuring the concentration of IS accumulation in the brain using HPLC analyses, the chronic administration of IS increased as the concentration increased compared to the control [9]. Additionally, a previous study showed dose-dependent apoptosis when treated with IS in the cortical and hippocampal cell lines [20]. We confirmed that an increase in cell death was observed when it was treated with IS on HT22 cells, a hippocampus cell line. Through this, it was expected that IS would directly act on the neuronal cells in uremia. EPA and DHA prevented cortisol-induced hippocampal injury by reducing oxidative damage [21]. Additionally, DHA protected hippocampal cells from oxidative damage [22]. In this study, EPA and DHA protected the HT22 cells from IS-induced cellular death.

ω-3 PUFA plays an important role in the brain, including in inflammatory response, immunity, cell growth, and tissue repair [23,24]. In the brain (hypothalamus, hippocampus, prefrontal cortex, and striatum) of diet-induced obese mice administered ω-3 PUFA (400 mg/kg/day) for 4 weeks, antioxidant defense increased and oxidative stress decreased [11]. The results confirmed that when ω-3 PUFA (4 g/kg) was administered once, it revealed a protective effect against uremic toxin in the hippocampus region. Furthermore, in the brain of the spinal cord injury model wherein ω-3 PUFA was administered orally, the mRNA expression of apoptotic markers such as Bcl2, Bax, and caspase-3 was decreased [25]. The results of this paper show that ω-3 PUFA plays a protective role against oxidative stress and neuronal cell apoptosis caused by uremic toxins.

Fat-1 mice have been reported to have attenuated brain damage in several brain damage models [26,27]. Hwang et al. reported that scopolamine-induced hippocampal cell apoptosis was attenuated in Fat-1 mice [26]. Luo et al. reported that apoptosis was attenuated in the hippocampus region in the multiple diffuse microinfarcts model of Fat-1 mice [27]. In this study, uremia-induced neuronal cell apoptosis was attenuated in Fat-1 mice.

PI(3)K-Akt signaling transduces mitogenic and metabolic signals to promote cell growth, proliferation, migration, and apoptosis [28]. A recent study revealed that the cerebral cortex of diabetic rats plays an important role in neuroprotection through the activation of PI(3)K-Akt signaling [29]. Additionally, Shi et al. reported that ω-3 PUFA attenuated LPS-induced nerve damage in neonatal rats via PI(3)K-Akt signaling. Furthermore, LPS-induced PC12 cell damage was reduced by treatment with ω-3 PUFA and the selective PI(3)K-Akt agonist 740Y-P, thus alleviating inhibition of cell proliferation, migration, and reduced apoptosis [30]. Similarly, this study showed that ω-3 PUFA plays a role in anti-cell death through PI(3)K-Akt signaling, thereby resulting in improved cell survival.

In conclusion, we showed that elevated uremic toxin increases ROS generation and apoptosis, and decreases neuron differentiation and proliferation in uremic mice. ω-3 PUFA increased neuronal cell survival in the brain injury induced by uremic toxin. Additionally, PI(3)K-Akt signaling is believed to be involved in these protective effects.

## 4. Materials and Methods

### 4.1. Cell Culture and Drug Treatment

HT22 cells, of the mouse hippocampal neuronal cell line, were incubated with Dulbecco’s modified Eagle’s medium (DMEM; WELGENE, Gyeongsan-si, South Korea) comprising 10% fetal bovine serum (Life Technologies Inc., Gaithersburg, MD, USA) and 1% Anti-Anti at 37 °C under 5% CO_2_. HT22 cells were plated and adhered (16–18 h) and treated with indoxyl sulfate (Alfa Aesar, Ward Hill, MA, USA) (1–70 mM) for 24 h. To determine the effects of drugs DHA and EPA (Sigma-Aldrich, St. Louis, MO, USA) on neurons, IS was treated for 1 h and then the drug was treated for 24 h. The cell viability was evaluated using a CCK-8 assay, according to the manufacturer’s protocol (Dojindo Molecular Technologies, Inc., Kumamoto, Japan). CCK-8 was added to each well, and then incubated for 3 h at 37 °C before measurement. Absorbance at 450 nm was detected using a microplate reader (Multiskan™ FC; Thermo Fisher Scientific, Waltham, MA, USA). Additionally, these cells were harvested and analyzed for molecules containing p-PI3K, p-PDK1, and p-Akt.

### 4.2. Animal Model

C57BL/6 mice (10 weeks old, male) were purchased from SAMTAKO Bio Korea (Gyounggido, South Korea) and Fat-1 transgenic mice (10 weeks old, male) were provided by Dr. Jing Xuan Kang of Harvard Medical School (Boston, MA, USA). All of the transgenic Fat-1 mice used were male and homozygous, and the absence or presence of the Fat-1 gene in each mouse was assured by genotyping. In the ω-3 PUFA model, IR was performed after the oral administration of ω-3 PUFA (4 g/kg) (Youngjin Pharmaceutical, Songpa-gu, South Korea) to C57BL/6 mice 24 h earlier. Food and water were freely consumed, and the mice were reared in a room maintained with a 12/12 h light/dark cycle. All of the animal experiments were conducted with the approval of the Animal Use and Care Committee at the Chungnam National University School of Medicine (202012A-CNU-162). The mice were divided into six groups: wild-type (wt) sham (*n* = 8), ω-3 PUFA sham (*n* = 8), Fat-1 sham (*n* = 8), wt IR (*n* = 20), ω-3 PUFA IR (*n* = 20), and Fat-1 IR (*n* = 20). The mice were anesthetized with an intraperitoneal injection of ketamine (60 mg/kg body mass) and xylazine (8 mg/kg). IR injury was performed as described previously [31]. After an abdominal incision, both renal pedicles were clamped bluntly. During the procedure, the clamps were removed 30 min after ischemia while maintaining body temperature of 37 °C with a heat pad for 40 min. Thereafter, all mice were sacrificed, and blood, kidney tissue, and brain tissue were collected three days after the procedure.

### 4.3. Blood and Tissue Preparation

Tissues were prepared as described previously [32]. Blood was collected from the inferior vena cava of the anesthetized mice. The blood was placed in the microcentrifuge tubes (4 °C). For blood urea nitrogen (BUN) and serum creatinine (s-Cr), the aliquots of serum were analyzed using a chemistry auto-analyzer, Toshiba 200FR (Toshiba Medical Systems Co., Tokyo, Japan). Kidney was fixed in 4% paraformaldehyde (4% PFA) at room temperature (RT) and then embedded in a Paraplast (Sherwood Medical, St. Louis, MO, USA) for light microscopy. The brain was perfused transcardially with 4% of PFA in PBS and the tissues were fixed in 4% PFA for 16–18 h at 4 °C. The brain was removed, dehydrated, embedded with OCT, frozen, and sectioned. The frozen section had a thickness of 30 μm.

### 4.4. Tissue Injury Score

The kidney tissue was made into paraffin blocks, cut into 4 µm, and attached to a slide glass. The sections were deparaffinized with xylene, stained with H&E, and examined under a microscope (Olympus BX51, Olympus, Tokyo, Japan). Five consecutive fields were examined at 200× magnification and tissue injury scores were averaged per slide. For the H&E sections, renal cortical vacuolization, proximal tubule simplification, renal cortical vacuolization, and peritubular/proximal tubule leukocyte infiltration were evaluated and scored as follows: normal = 0, mild injury = 1, moderate injury = 2, and severe injury = 3. The injury scoring in H&E staining was evaluated by an experienced pathologist in a blind fashion.

### 4.5. Western Blot Analysis

Briefly, the proteins were extracted with buffer containing 1M PBS, 5Mm EDTA, and 0.5% Triton X-100. After centrifugation (13,000 rpm for 10 min, 4 °C), the supernatant was collected for Western blot analyses. Protein (20 µg/lane) was electrophoresed on 10–15% SDS gel, and then transferred to polyvinylidene fluoride (PVDF) membranes. The membranes were blocked with 5% non-fat dry milk for 1 h at RT and then incubated with primary antibodies against α-tubulin (1:1000, Cell Signaling Technology, Danvers, MA, USA), Bcl2, PARP and Bad (1:1000, 1:1000, and 1:1000, respectively, Sigma-Aldrich, St. Louis, MO, USA), p-PI3K, p-PDK1, p-Akt and Bax, and cleaved caspase-3 (1:1000, 1:1000, 1:1000 and 1:1000, and 1:1000, respectively, Cell Signaling Technology, Danvers, MA, USA) at 4 °C overnight. The membranes were incubated with HRP-conjugated anti-rabbit IgG secondary antibodies (1:2000, Abfrontier Co., Ltd., Seoul, Korea) and HRP-conjugated anti-mouse IgG secondary antibodies (1:2000, Abfrontier Co., Ltd., Seoul, Korea) for 2 h at RT. The protein bands were visualized using a chemiluminescence detection kit (Thermo Scientific, South Logan, UT, USA). The same membranes were subsequently used for α-tubulin immune detection, and equal protein loading was ensured. The optical density for quantification was obtained using Gel-Pro Analyzer version 3.1 (Media Cybernetics, Silver Spring, MD, USA).

### 4.6. Terminal Deoxynucleotidyl Transferase dUTP Nick End Labeling Staining

Frozen sections (30 μm) were incubated in a 60 °C oven for 2 h and incubated in 4% PFA for 15 min at RT. Endogenous peroxidase was blocked with 3% hydrogen peroxide diluted in PBS. The sections were experimented for TUNEL staining using an In Situ Cell Death Detection Kit (*Fluorescein*) (11684795910, Roche), following the manufacturer’s recommendations. The TUNEL-positive cells were identified with fluorescent signals using a fluorescent microscope. The apoptosis cells were counted in the subgranular zone of the hippocampal dentate gyrus (DG) in both hemispheres on one coronal section of the mice brain. To semi-quantitatively evaluate apoptosis, five different microscopic fields in the subgranular zone of the hippocampal dentate gyrus (DG) were selected randomly at 200× magnification. TUNEL positive cells were counted as the number of positively staining cells per high power field (HPF, 200× magnification). Four mice brains per group were analyzed.

### 4.7. Immunofluorescence Staining

The section was incubated with primary antibodies against Ki67 (1:200, Abcam, Cambridge, UK) and neuronal nuclei (NeuN) (1:100, ABN90, EMD Millipore, Burlington, MA, USA) at 4 °C overnight. It was further incubated with secondary antibodies against goat Alexa Fluor^®®^ and Alexa Fluor^®®^ 488 conjugated anti-mouse antibody at RT in the dark for 2 h, cover-slipped with Fluoroshield™ with 4′,6-diamidino-2-phenylindole (DAPI), and then observed and imaged under a fluorescent microscope. The number of ki67 or neu1-positive cells against the subgranular zone of the hippocampal dentate gyrus (DG) in both hemispheres on one coronal section of the mice brain was counted. To semi-quantitatively evaluate Ki67 and NeuN, five different microscopic fields in the subgranular zone of the hippocampal dentate gyrus (DG) were selected randomly at 200× magnification. Ki67- and NeuN-positive cells were counted as the number of positively staining cells per high power field (HPF, 200× magnification). Four mice brains per group were analyzed.

### 4.8. Statistical Analysis

All data is expressed as mean ± SD. Multiple comparisons among groups were analyzed using one-way ANOVA with a post hoc Bonferroni correction. We used SPSS software (ver. 20.0 for Windows; SPSS, Inc., Chicago, IL, USA). The differences among the groups were considered significant at *p* < 0.05.

## Data Availability

Not applicable.
